# Resting state EEG power spectrum and functional connectivity in autism: a cross-sectional analysis

**DOI:** 10.1186/s13229-022-00500-x

**Published:** 2022-05-18

**Authors:** Pilar Garcés, Sarah Baumeister, Luke Mason, Christopher H. Chatham, Stefan Holiga, Juergen Dukart, Emily J. H. Jones, Tobias Banaschewski, Simon Baron-Cohen, Sven Bölte, Jan K. Buitelaar, Sarah Durston, Bob Oranje, Antonio M. Persico, Christian F. Beckmann, Thomas Bougeron, Flavio Dell’Acqua, Christine Ecker, Carolin Moessnang, Tony Charman, Julian Tillmann, Declan G. M. Murphy, Mark Johnson, Eva Loth, Daniel Brandeis, Joerg F. Hipp, The EU-AIMS LEAP group authorship, Jumana Ahmad, Sara Ambrosino, Bonnie Auyeung, Tobias Banaschewski, Simon Baron-Cohen, Sarah Baumeister, Christian F. Beckmann, Sven Bölte, Thomas Bourgeron, Carsten Bours, Michael Brammer, Daniel Brandeis, Claudia Brogna, Yvette de Bruijn, Jan K. Buitelaar, Bhismadev Chakrabarti, Tony Charman, Ineke Cornelissen, Daisy Crawley, Flavio Dell’Acqua, Guillaume Dumas, Sarah Durston, Christine Ecker, Jessica Faulkner, Vincent Frouin, Pilar Garcés, David Goyard, Lindsay Ham, Hannah Hayward, Joerg Hipp, Rosemary Holt, Mark H. Johnson, Emily J. H. Jones, Prantik Kundu, Meng-Chuan Lai, Xavier Liogier D’ ardhuy, Michael V. Lombardo, Eva Loth, David J. Lythgoe, René Mandl, Andre Marquand, Luke Mason, Maarten Mennes, Andreas Meyer-Lindenberg, Carolin Moessnang, Nico Mueller, Declan G. M. Murphy, Bethany Oakley, Laurence O’Dwyer, Marianne Oldehinkel, Bob Oranje, Gahan Pandina, Antonio M. Persico, Barbara Ruggeri, Amber Ruigrok, Jessica Sabet, Roberto Sacco, Antonia San José Cáceres, Emily Simonoff, Will Spooren, Julian Tillmann, Roberto Toro, Heike Tost, Jack Waldman, Steve C. R. Williams, Caroline Wooldridge, Marcel P. Zwiers

**Affiliations:** 1 Roche Pharma Research and Early Development, Neuroscience and Rare Diseases, Roche Innovation Center Basel, Basel, Switzerland; 2 Department of Child and Adolescent Psychiatry and Psychotherapy, Central Institute of Mental Health, Medical Faculty Mannheim, Heidelberg University, Mannheim, Germany; 3 Department of Psychological Sciences, Centre for Brain and Cognitive Development, Birkbeck, University of London, London, UK; 4 Institute of Neuroscience and Medicine, Brain and Behaviour (INM-7), Research Centre Jülich, Jülich, Germany; 5 Medical Faculty, Institute of Systems Neuroscience, Heinrich Heine University Düsseldorf, Düsseldorf, Germany; 6 Department of Psychiatry, Autism Research Centre, University of Cambridge, Cambridge, UK; 7 Department of Women’s and Children’s Health, Center of Neurodevelopmental Disorders (KIND), Centre for Psychiatry Research, Karolinska Institutet and Child and Adolescent Psychiatry, Stockholm Health Care Services, Region Stockholm, Stockholm, Sweden; 8 Curtin Autism Research Group, Curtin School of Allied Health, Curtin University, Perth, WA Australia; 9 Department of Cognitive Neuroscience, Donders Institute for Brain, Cognition and Behaviour, Radboudumc, Nijmegen, The Netherlands; 10 Brain Center Rudolf Magnus, University Medical Center Utrecht, Utrecht, The Netherlands; 11 Interdepartmental Program “Autism 0-90”, “G. Martino” University Hospital, University of Messina, Messina, Italy; 12 Human Genetics and Cognitive Functions, Institut Pasteur, UMR3571 CNRS, Université de Paris, Paris, France; 13 Institute of Psychiatry, Psychology and Neuroscience, King’s College, London, UK; 14 Department of Child and Adolescent Psychiatry, Psychosomatics and Psychotherapy, University Hospital, Goethe University, Frankfurt am Main, Germany; 15 Department of Child and Adolescent Psychiatry and Psychotherapy, Psychiatric Hospital, University of Zurich, Zurich, Switzerland; 16 Neuroscience Center Zurich, University and ETH Zurich, Zurich, Switzerland

**Keywords:** Autism spectrum disorder, EEG, Resting state, Power spectrum, Functional connectivity

## Abstract

**Background:**

Understanding the development of the neuronal circuitry underlying autism spectrum disorder (ASD) is critical to shed light into its etiology and for the development of treatment options. Resting state EEG provides a window into spontaneous local and long-range neuronal synchronization and has been investigated in many ASD studies, but results are inconsistent. Unbiased investigation in large and comprehensive samples focusing on replicability is needed.

**Methods:**

We quantified resting state EEG alpha peak metrics, power spectrum (PS, 2–32 Hz) and functional connectivity (FC) in 411 children, adolescents and adults (*n* = 212 ASD, *n* = 199 neurotypicals [NT], all with IQ > 75). We performed analyses in source-space using individual head models derived from the participants’ MRIs. We tested for differences in mean and variance between the ASD and NT groups for both PS and FC using linear mixed effects models accounting for age, sex, IQ and site effects. Then, we used machine learning to assess whether a multivariate combination of EEG features could better separate ASD and NT participants. All analyses were embedded within a train-validation approach (70%–30% split).

**Results:**

In the training dataset, we found an interaction between age and group for the reactivity to eye opening (*p* = .042 uncorrected), and a significant but weak multivariate ASD vs. NT classification performance for PS and FC (sensitivity 0.52–0.62, specificity 0.59–0.73). None of these findings replicated significantly in the validation dataset, although the effect size in the validation dataset overlapped with the prediction interval from the training dataset.

**Limitations:**

The statistical power to detect weak effects—of the magnitude of those found in the training dataset—in the validation dataset is small, and we cannot fully conclude on the reproducibility of the training dataset’s effects.

**Conclusions:**

This suggests that PS and FC values in ASD and NT have a strong overlap, and that differences between both groups (in both mean and variance) have, at best, a small effect size. Larger studies would be needed to investigate and replicate such potential effects.

**Supplementary Information:**

The online version contains supplementary material available at 10.1186/s13229-022-00500-x.

## Background

Autism spectrum disorder (ASD) is a prevalent neurodevelopmental condition, affecting around 1 in 54 children [1]. It is diagnosed in the presence of alterations in social communication, social interaction and restricted and repetitive behaviors and interests [2]. ASD is heterogeneous with diverse clinical presentations and frequent comorbidities including epilepsy, attention-deficit/hyperactivity disorder, anxiety and depression [3]. Currently, the pathophysiology of ASD is unclear. In particular, neuronal mechanisms underlying idiopathic ASD remain largely unknown [2]. Establishing potential differences in brain activity between ASD and NT and heterogeneities within the ASD population would be an important step to shed light into the etiology of the condition and facilitate the development of interventions.

Electrophysiological techniques such as EEG (electroencephalography) or MEG (magnetoencephalography) are suited to investigate macroscopic neuronal circuit function and maturation. They are noninvasive and direct measures of neuronal activity with a high temporal resolution enabling the evaluation of brain dynamics and brain rhythms. The value of EEG in ASD was recently highlighted by several investigations showing a consistent deviation of event-related activity in response to presentation of human faces [4], i.e., the N170 latency, and subsequent efforts to develop it into a qualified biomarker for use in ASD clinical trials [5]. Besides the event-locked EEG response to sensory stimuli, resting state EEG—that is the EEG signal in the absence of any specific task—could provide important insights into brain circuitry associated with ASD. In particular, the resting state power spectrum (PS), quantifying local synchronization within brain regions, and functional connectivity (FC) measures, quantifying long-range interactions between distant brain areas, are key characteristics of the resting-state EEG [6, 7].

Many studies have evaluated resting state EEG/MEG PS and FC in ASD across brain rhythms, from the delta to the gamma range, and some focusing on the most prominent resting state rhythm: alpha. However, no clear picture has emerged yet. Various theories for PS alterations in ASD have been put forward, including a U-shaped profile with excessive power in low and high frequencies [8], but many findings are in conflict to each other. For example, for spectral power in the alpha band, there are various reports of increase [9–11], decrease [12–14] and no effects [15, 16] in ASD compared to NT (neurotypicals). O’Reilly et al. performed a systematic review of the FC ASD literature [17]. Although a meta-analysis could not be performed due to large variability in methodology and samples across the 52 reports included, the authors concluded that there was a trend for a decrease of long-range FC in ASD—even as the frequency bands and brain/scalp regions of these effects were unclear, and despite several studies finding either no difference or even increased FC in ASD [18–20]. The inconsistencies of the results across studies could be driven by false positives, publication bias, small sample sizes, age effects, heterogeneity in the ASD population and co-occurring conditions such as intellectual disability, among other factors. These ambiguities highlight the need for a hypothesis-free evaluation of PS and FC in a large sample of ASD, focusing on replicability and generalizability.

Here, we evaluated resting state EEG PS and FC in ASD (*n* = 212) and NT (*n* = 199) children, adolescents and adults (all with IQ > 75), using the baseline visit of the Longitudinal European Autism Project (LEAP) for a cross-sectional analysis [21, 22]. We focused on diagnostic effects and compared ASD and NT groups. In light of the conflicting literature, we performed a hypothesis-free evaluation of PS and FC without restriction on the frequencies, brain/scalp areas of interest, and directionality. We adopted a flexible analytical approach testing various models and evaluated the generalizability of our findings. To this end, we used cluster-based permutation statistics, which optimally account for the dimensionality of the data, to control for multiple testing across source locations and frequencies within specific models. Importantly, we used a validation dataset to test any findings from the main analysis and thereby account for possible false positives due to testing of multiple models.

## Materials and methods

### Study design and EEG acquisition

The resting state EEG data analyzed here were recorded as part of EU-AIMS LEAP. A complete description of the study design and clinical characterization of the participants can be found elsewhere [21, 22]. Briefly, participants with ASD were recruited based on an existing clinical diagnosis of ASD according to DSM-IV, DSM-IV-TR, DSM-5 or ICD-10 criteria and all participants were between 6 and 32 years. ASD symptomatology was assessed with various instruments including the Autism Diagnostic Observation Schedule-2 (ADOS-2) and the Autism Diagnostic Interview-Revised (ADI-R), but participants were not excluded based on ADOS-2 and ADI-R scores. In this work, we only include autistic individuals with average to high intellectual capacities (IQ > 75) who successfully completed the resting state EEG recording (211 NT and 242 ASD).

Four minutes of resting state EEG were recorded per participant (2 min with eyes open, 2 min with eyes closed). To optimize participant compliance, resting state was acquired in 30 s blocks, alternating eyes open (fixating a physical hourglass) and eyes closed. Data were acquired at five sites: Central Institute of Mental Health (CIMH, Mannheim, Germany), King’s College London (KCL, United Kingdom), University Nijmegen Medical Centre (RUNMC, Netherlands), University Campus BioMedico (UCBM, Rome, Italy) and University Medical Centre Utrecht (UMCU, Netherlands). The following EEG systems were employed: Brainvision (CIMH, KCL, RUNMC), Biosemi (UMCU) and Micromed (UCBM), with sampling frequencies of 5000 Hz (KCL, RUNMC), 2048 Hz (UMCU), 2000 Hz (CIMH) and 256–1000 Hz (UCBM). All sites used 10–20 layout caps, with 60–70 electrodes.

### EEG data processing

#### Preprocessing

EEG signals were resampled to 1000 Hz and then band pass filtered to [1–32] Hz with a finite impulse response filter of order 2000 (using 2 s of padding at each edge of a resting state block). Higher frequencies (in the gamma range) were not included in the analysis to avoid substantial contamination with muscle activity [23]. Only data from 61 electrodes common to most contributing sites were retained for subsequent analyses. Preprocessing was performed manually and blinded to the participants diagnosis and clinical information, following these sequential steps: (a) Eliminate bad channels, (b) locate and discard sections with large transient artifacts resulting, e.g., from muscle bursts or movements, (c) perform independent component analysis with fastICA (http://www.cis.hut.fi/projects/ica/fastica/, [24]), (d) detect artefactual components (capturing: ocular, muscular, cardiac or other artifacts), (e) eliminate the contribution of artefactual components, (f) iterate points (b-e) if necessary, (g) interpolate the bad channels that were eliminated in (a), and (h) re-reference signals to the average across all channels (average reference).

Participants were excluded from subsequent analyses if they met any of the following criteria: (1) less than 15 clean 2.5 s epochs in the eyes-open or the eyes-closed condition (11 participants), (2) less than 51 usable channels or more than 3 neighboring channels eliminated (9 further participants), (3) number of good channels minus number of artefactual independent components smaller than 35 (threshold selected visually when inspecting the distribution of values, 6 further participants). Following these criteria, 26 recordings (18 ASD and 8 NT) were discarded, resulting in a sample size of 224 ASD and 203 NT.

#### Summary resting state alpha measures

We inspected the alpha peak of each participant. Alpha is the most prominent rhythm in the resting EEG and could reveal if there was a shift in frequencies between the ASD and the NT groups (e.g., one group having alpha at higher frequencies than the other). Power spectra were computed for each channel and condition (eyes open and eyes closed) with fast Fourier transform and 2.5 s Hanning windows with 75% overlap. Alpha peaks in the [6, 13] Hz range were detected automatically by fitting a Gaussian over a power law background to the average eyes closed power spectrum over occipital channels O1, O2, Oz, PO4, PO3 and POz, following [25]. A single alpha peak frequency was derived per participant (and not separate ones for eyes open and eyes closed). Automatic fits were verified by visual inspection and refined for 11 out of 224 ASD and 10 out of 203 NT participants. Seven participants with no clear alpha peak were discarded from subsequent analyses, yielding a total of 218 ASD and 202 NT participants.

The following summary alpha measures were derived from the alpha peak: (1) alpha peak frequency $f_{{\text{p}}}$, (2) alpha power, defined as the absolute power in the range $\left[ {f_{{\text{p}}} - 2 {\text{Hz}}, f_{{\text{p}}} + 2 {\text{Hz}}} \right]$ over occipital sensors (O1, O2, Oz, PO4, PO3, POz), (3) reactivity to eye opening, defined as $R = 1 - \frac{{P_{{{\text{EO}}}} }}{{P_{{{\text{EC}}}} }}$, where $P_{{{\text{EO}}}}$ and $P_{{{\text{EC}}}}$ are absolute power values defined previously for the eyes-open and the eyes-closed condition.

#### Source reconstruction

Head models with realistic geometry were built from individual’s T1 weighted MRIs. Details on MRI acquisition can be found in [26]. T1-weighted MRIs were segmented with SPM12 [27] into gray matter, white matter, cerebrospinal fluid, bone, soft tissue and air. Then, these probabilistic images were smoothed (5 mm FWHM), thresholded and resliced to produce binary masks of 2 mm × 2 mm × 2 mm resolution for three tissue types: brain (including gray matter, white matter and cerebrospinal fluid), skull and scalp. These binary masks were transformed to hexahedral meshes with FieldTrip [28]. All three considered tissue types were assumed to have homogeneous and isotropic conductivity: 330 mS/m for the brain and scalp [29], and an age-dependent skull conductivity of 3.958 + 62.77*exp(− 0.2404*age[years]) mS/m, in line with the BESA (BESA, Gräfelfing, Germany) recommended conductivity ratios. Of note, the conductivity of cerebrospinal fluid is higher than that of gray or white matter [30], so setting the conductivity of the brain compartment as a constant and isotropic value is a simplification, but the 3-tissue model has led to similar spatial accuracy than a model including a separate CSF tissue in a previous publication [31]. Segmentations were visually inspected, and for 9 participants head models could not be built (because of either no MRI or no clean segmentations). As a consequence, subsequent analyses included 212 ASD and 199 NT participants.

The forward model was derived with FieldTrip and SimBio [32]. 365 source locations of interest were defined in gray matter in MNI space, following a 3D cubic diamond grid with 1.5 cm spacing. Source positions were transformed from MNI to each subject’s individual space with a nonlinear transformation obtained with SPM12. Electrode positions were determined by transforming standard MNI positions to subject’s space with this same transformation and projecting to the scalp surface. Source time series were estimated with linearly constrained minimum variance beamformer, using a regularization of 5% of the average trace of the covariance matrix, a common filter for resting state eyes open and eyes closed and projecting the source time series into the direction of maximal power [33].

#### Power spectrum (PS)

Power spectra were computed for each sensor, source and condition: Time series (source or sensor) were convolved with Morlet wavelets of 0.6 octave frequency resolution (*f*/*σ*_f_ = 4.88) and $5 \cdot \sigma_{t}$ window length, with 90% overlap between windows, for frequencies $f\; = \;2^{1:0.15:5}$ Hz (frequencies from 2 to 32 Hz in increments of 0.15 in the exponent value). Since beamformer reconstructions suffer from power bias especially for deeper sources [34], source space PS values were normalized with the overall power over the [2 32] Hz range to produce relative PS values. Of note, all subsequent analyses were performed with source space data, except for a control analysis using absolute power at the sensor level.

#### Functional connectivity (FC)

The 365 sources (see above) were grouped into 50 regions of interests (ROIs) (25 symmetrical ROIs per hemisphere, obtained through k-means clustering of the source positions, leading to 4–13 sources per ROI). The representative time series for a given ROI was defined as the first principal component of all the source time series in this ROI. For each combination of ROIs and frequency, functional connectivity (FC) was quantified with orthogonalized power correlations (orthPowCorr) and weighted phase lag index (wPLI), which evaluate complementary phase and amplitude synchronization while discarding zero-lag synchronization which could be driven by volume conduction. OrthPowCorr and wPLI were implemented following [35] and [36], respectively. In order to avoid introducing sample size-related bias in wPLI, resting state data were segmented into non-overlapping 2.5 s clean epochs, and wPLI was estimated across 15 epochs. These 15 epochs were selected randomly from all the clean epochs from a given subject and condition, this process was repeated 100 times, and values were averaged across the 100 repetitions. Control analyses with other FC metrics are performed using direct power correlations (PowCorr), coherence (COH), imaginary coherence (iCOH) and phase locking value (PLV), following [6, 35, 37]. More details on the FC metrics can be found in Additional file 1.

### Participants

The statistical analyses reported in subsequent sections were performed using 212 ASD and 199 NT participants, after discarding 42 participants because of unsuccessful preprocessing, lack of clear alpha peak or no available head model (26, 7 and 9 participants, respectively, as detailed in previous sections). The groups of included and excluded autistic participants differed significantly, at the uncorrected group level, on Vineland (Adaptive Behaviors subscale scores) and ADOS-2 (Restricted and Repetitive Behaviours scores), with higher symptomatology in the excluded group (see Additional file 1 Table S1 for more details). This might be expected since participants with higher symptom expression are less likely to be able to comply with instructions, and more likely to move or generate artifacts during the EEG and MRI acquisition sessions. Table 1 summarizes the main demographics and clinical characteristics of the included participants. Age and sex did not differ between groups (*p* = 0.71 and *p* = 0.24, respectively). Similarly, the amount of clean data did not differ significantly (*p* > 0.15) between groups for eyes open (ASD: mean 102 s, SD 13 s, range 55–120 s; NT: mean 104 s, SD 14 s, range 46–120 s) or eyes closed (ASD: mean 104 s, SD 13 s, range 60–125 s, NT: mean 106 s, SD 13 s, range 58–120 s) conditions.

**Table 1:** Overview of clinical and demographic characteristics of the participants included in the statistical analyses

	ASD	NT	Group differences
*n*	212	199	
Age (years)	16.6 ± 5.7 [6.7–30.3]	16.8 ± 6.0 [6.9–30.8]	*d* = − 0.04 *p* = 0.71
Child/Adol/Adult	53/76/83	54/69/76	
IQ	104.0 ± 14.5 [75.6–148.0]	107.9 ± 13.1 [75.6–142.0]	*d* = − 0.28 *p* = 0.0045
Sex (M/F)	153/59	132/67	*p* = 0.24
ADI social	15.3 ± 6.9 [0–29] (*n* = 202)		
ADI-R communication	12.5 ± 5.6 [0–26] (*n* = 202)		
ADI-R RRB	4.2 ± 2.8 [0–12] (*n* = 202)		
ADOS-2 Social Affect CSS	5.9 ± 2.6 [1–10] (*n* = 209)		
ADOS-2 RRB CSS	4.6 ± 2.6 [1–10] (*n* = 209)		
ADOS-2 Total CSS	5.1 ± 2.7 [1–10] (*n* = 209)		
VABS	74.4 ± 14.2 [20–121] (*n* = 174)	103.2 ± 11.7 [70–127] (*n* = 48)	*d* = − 2.21 *p* < 0.0001
SRS-2	84.6 ± 31.2 [20–163] (*n* = 193)	25.0 ± 16.5 [1–94] (*n* = 168)	*d* = 2.39 *p* < 0.0001
Medication (%)	36.9% (*n* = 198)	5.6% (*n* = 179)	*p* < 0.0001

*Child* Children (age 6–11), *Adol* Adolescents (age 12–17), and adults are aged 18 years and above, IQ—full scale IQ, *M* male, *F* female, ADI social, ADI communication and ADI RRB refer to the Social, Communication and Restricted and Repetitive Behaviours total domain scores of the ADI-R (Autism Diagnostic Interview-Revised). ADOS Social Affect, ADOS RRB and ADOS Total refer to the Social Affect, Restricted and Repetitive Behaviours and Total calibrated severity scores in ADOS-2. VABS refers to the Vineland Adaptive Behavior Second Edition Adaptive Behavior Composite standard score. SRS-2 refers to the Social Responsiveness Scale-2 Total score (combined parent- and self-report). Medication refers to brain active medication (antidepressants, antimigraine, antipsychotics, anxiolytics, hypnotics, sedatives, psychostimulants, analgesics, etc.). Values from numerical variables are reported as mean ± standard deviation [min–max]. *P* values for the group effects are indicated (*t* test for continuous variables, Fisher’s exact test for categorical variables) along with Cohen’s d

### Statistical analysis: data-driven exploration followed by validation in independent sample

**Fig. 1:**
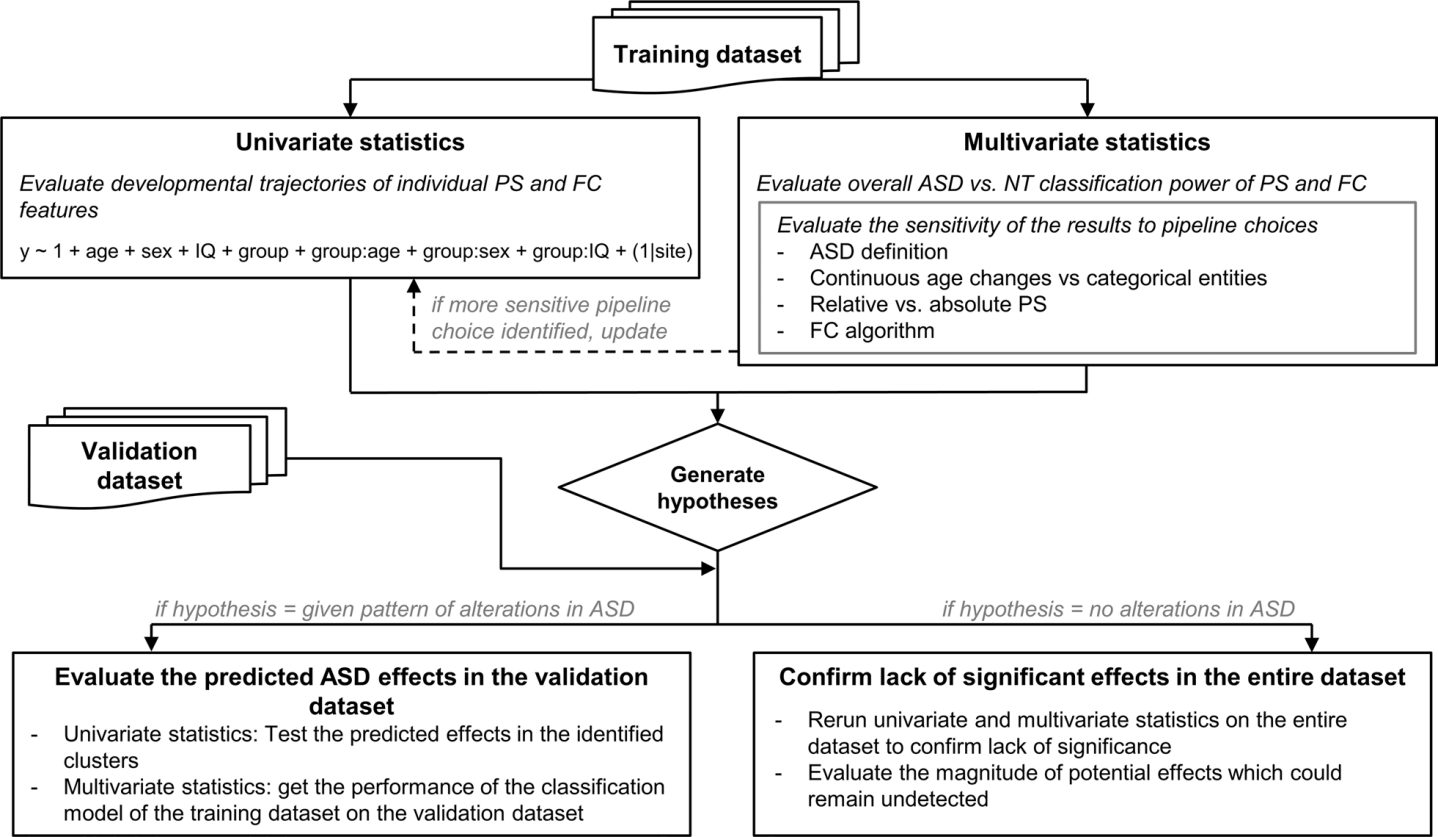
Overview of the statistical analysis approach. Univariate and multivariate statistics were performed in the training dataset, as well as control comparisons to evaluate the sensitivity of the results to pipeline choices. From this, concrete hypotheses are generated and tested in the validation dataset

We opted for a hypothesis-free statistical analysis rather than a targeted approach testing concrete hypotheses given that the ASD resting state literature is contradictory and a variety of effects across brain rhythms and brain regions have been reported. We assessed potential power spectra and FC alterations across frequencies and brain regions. Furthermore, we evaluated the generalizability of our outcomes by evaluating the impact of several analyses choices such as FC metric, statistical models, or ASD definition. In order to do so, while maintaining high statistical flexibility, we followed a train/validation approach. That is, we separated the dataset into training (70% of the participants: 147 ASD and 140 NT) and validation datasets (30% of the participants: 65 ASD and 59 NT). The 30%–70% split was performed using stratified randomization by site, age group (children, adolescents, adults) and diagnosis. The univariate and multivariate statistics described in the following sections were performed using the training dataset exclusively. Clear hypotheses were derived from the analyses in the training dataset, which were then tested in the validation dataset. To assess the consistency between results in the training and validation datasets, prediction intervals were calculated for the univariate analysis. They estimate the range of values that can be expected in a replication due to chance and were calculated following [38] for comparing effect sizes. An overview of the statistical approach is shown in Fig. 1. Clinical and demographic characteristics of the participants in the training and validation datasets can be found in Additional file 1: Tables S2 and S3.

#### Univariate statistics

Each summary alpha measure, PS and FC feature was submitted to the following linear mixed effects (LME) models:

$$\begin{array}{*{20}l} {y\sim 1 + {\text{age}} + {\text{sex}} + {\text{IQ}} + \left( {1|{\text{site}}} \right),{\text{homoscedastic residual errors}}} \hfill & {\left( {{\text{model }}1} \right)} \hfill \\ \begin{gathered} y\sim 1 + {\text{age}} + {\text{sex}} + {\text{IQ}} + {\text{group}} + {\text{group}}:{\text{age}} + {\text{group}}:{\text{sex}} + {\text{group}}:{\text{IQ}} + \left( {1|{\text{site}}} \right), \hfill \\ {\text{homoscedastic residual errors}} \hfill \\ \end{gathered} \hfill & {\left( {{\text{model }}2} \right)} \hfill \\ \begin{gathered} y\sim 1 + {\text{age}} + {\text{sex}} + {\text{iq}} + {\text{group}} + {\text{group}}:{\text{age}} + {\text{group}}:{\text{sex}} + {\text{group}}:{\text{IQ}} + \left( {1|{\text{site}}} \right), \hfill \\ {\text{heteroscedastic residual errors}} \hfill \\ \end{gathered} \hfill & {\left( {{\text{model }}3} \right)} \hfill \\ \end{array}$$

All models were fit with the nlme package in R version 3.4.0 [39], using maximum likelihood estimation. Models 1 and 2 assumed homoscedastic residual errors, while model 3 allowed for different residual variances in the ASD and NT groups. Model 3 is the full model shown in Fig. 1. Age and IQ were mean centered and variance normalized before entering the statistical models. The statistical significance of group differences in mean was determined through the log-likelihood ratio between models 2 and 1. Log-likelihood tests have been reported to be “anticonservative”: They may output *p* values that are lower than the nominal *p* values [40]. Therefore, significant group effects were confirmed by using *F*-tests on the individual parameters after fitting the models with a restricted maximum likelihood method [39]. The statistical significance of group differences in variance was determined through the log-likelihood ratio between models 3 and 2. The following transformations were applied before LME in order to convert the feature distributions to normality: $x \to \log \left( x \right)$ (absolute power), $x \to x^{4}$ (alpha reactivity), $x \to a\tanh \left( x \right)$ (orthPowCorr) and $x \to x^{0.11}$ (wPLI). The optimal transformations to normality were obtained by evaluating the Kolmogorov–Smirnov goodness-of-fit test for several families of transformations. For information on site and medication effects, refer to Additional file 1.

Cluster-based permutation tests were used to control for multiple comparisons for the PS and FC measures, following [41]. First, clusters of frequencies and electrodes/sources/links with significant effects (*p* < 0.05, uncorrected) were created. For PS, two sources/electrodes were considered neighbors if they were spatially adjacent. For FC, two links were considered neighbors if they had one common node and one spatially adjacent node. (Two ROIs are considered adjacent if they have at least one pair of neighboring sources.) Then, the cluster size was compared with the null-hypothesis distribution derived from the maximal cluster sizes obtained in randomized datasets (2000 randomizations were performed by randomizing group labels exclusively). *P* values were defined as the proportion of randomizations, which yielded a larger cluster size than the original dataset.

#### Multivariate statistics

To test whether a multivariate combination of PS and FC features, respectively, can discriminate ASD from NT, we used machine learning techniques. Before subjecting the data to the multivariate analyses, the effects of age, sex, IQ and site were removed by building PS and FC datasets containing the residuals of the LME models y ~ 1 + age + sex + IQ + (1|site) trained on individual PS and FC features. The PS dataset consists in the space x frequency PS values for eyes-open and eyes-closed condition (19,710 features). The FC dataset contains FC strength across links, frequencies and conditions (66,150 features). Principal component analysis was applied to the PS and FC datasets (matrices of EEG features x subjects), and the principal components explaining at least 98% of the variance were used as input for the classification algorithms (160, 392 and 389 components for PS, OrthPowCorr and wPLI, respectively; each of these principal components is a linear combination of EEG features).

To date, a plethora of classification approaches has been introduced and proven useful [42]. The performance of the models depends greatly on the feature selection approach, classification algorithm and hyperparameter values. Here, we use three different classification approaches that have been successfully used in previous neuroimaging studies and are implemented with scikit-learn [43]: First, we use the L2-penalized support vector classifier (linSVC) which is one of the most common approaches in neuroimaging [44, 45]. Second, we estimate an elastic net logistic regression, which combines L1 and L2 regularization and could perform better if only a small number of features was sufficient for the differentiation between two groups [45, 46]. Third, we combine a Boruta feature selection (based on random forests) with a radial basis kernel support vector classifier, which can detect nonlinear patterns differentiating between two groups [47]. Classifier hyperparameters were tuned using grid search (sklearn.model_selection.GridSearchCV) and nested tenfold cross-validation. The harmonic mean of sensitivity and specificity (also known as S1 score) was employed as the scoring metric, since it prevents algorithmic convergence into a trivial single class prediction in unbalanced datasets [48]. Further details on the classification models employed can be found in Additional file 1: Table S4. Repeated random splits [49] were used to evaluate the cross-validation performance of the classification models (15 random splits with 20% left-out data, using sklearn.model_selection.StratifiedShuffleSplit) within the training set. The significance of the classification performance of the classifiers was assessed by comparing the scoring metric in the original PS and FC datasets with the corresponding values obtained when training the classifiers in datasets with randomized group labels (1000 randomizations).

## Results

### Univariate statistics

**Fig. 2:**
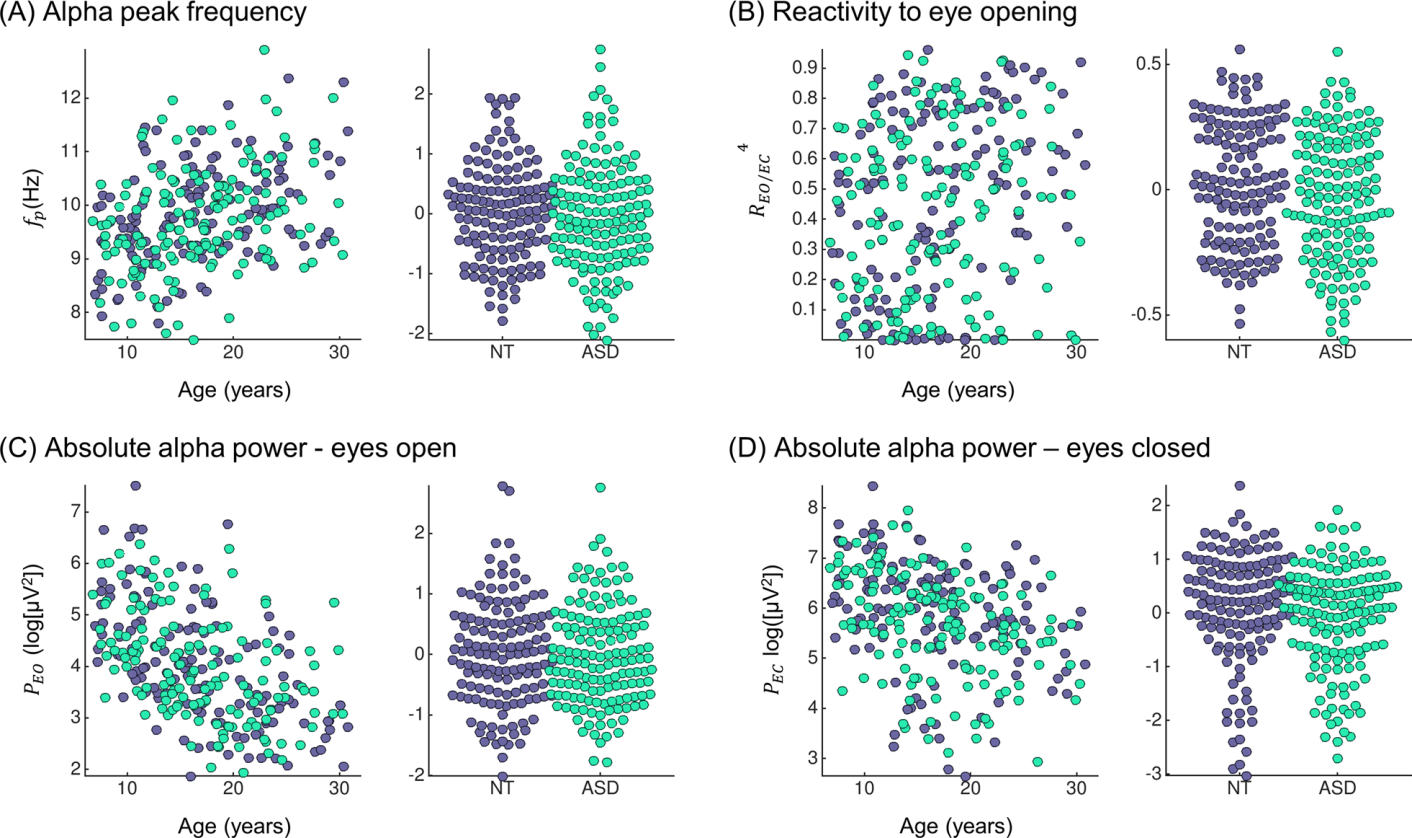
Alpha peak measures. For each measure, the scatter plot of the raw values as a function of age is shown in the left side and the residuals of the linear mixed effects model y ~ 1 + age + sex + IQ + (1|site) on the right side. All plots derived from the training dataset (147 ASD and 140 NT)

First, we studied properties of the EEG alpha peak, the most prominent feature of the awake human EEG. As expected from the literature, both the alpha peak frequency and absolute power significantly changed with age, with increasing peak frequency and decreasing power (Fig. 2, *p* values for age coefficient: alpha peak frequency *p* = 7.0·10^−11^, absolute power eyes open *p* = 3.2·10^−17^, absolute power eyes closed *p* = 1.9·10^−9^). We next investigated whether the groups differed in mean or variance. (A higher variance in the ASD group could be indicative of increased variability or heterogeneity.) The mean reactivity to eye opening differed between the ASD and NT groups (log-likelihood test *p* = 0.042): There was a significant interaction between group and age (*F*-test, *p* = 0.048): The age-related increases in reactivity were stronger in the NT than in the ASD cohort. We found no significant differences between ASD and NT for alpha peak frequency or absolute power in the training sample and no difference between variances for any measure (*p* > 0.12, Table 2).

**Table 2:** *P* values from the univariate statistics (training dataset, 147 ASD and 140 NT)

	Differences in mean		Differences in variance	
Alpha peak frequency	0.77 (*d* = − 0.11)		0.12	
Reactivity to eye opening	**0.042 (*d* = ** − **0.18)**		0.76	

	Eyes open	Eyes closed	Eyes open	Eyes closed
Absolute alpha power	0.43 (*d* = − 0.05)	0.39 (*d* = − 0.20)	0.37	0.28
Power spectrum	0.72 (*d* = − 0.05)	0.91 (*d* = 0.27)	0.53	0.92
wPLI	0.90 (*d* = − 0.24)	0.98 (*d* = − 0.05)	0.89	0.23
OrthPowCorr	0.59 (*d* = − 0.15)	0.91 (*d* = 0.12)	0.39	0.85

*P* values were obtained after comparing the log-likelihoods of the three linear mixed effects models (see Materials and Methods). For power spectrum, wPLI and OrthPowCorr, *p* values were derived from cluster-based permutation tests. Cohen’s *d* values are given for reference after the *p* values, but note that they do not directly reflect statistical significance, since they were computed with the raw EEG parameters and do not correct for any covariates. wPLI: Weighted phase lag index. OrthPowCorr: Orthogonalized power correlations. *P* values under 0.05 are highlighted in bold

Next, we investigated source space spectral power and functional connectivity. As expected from the literature, both PS and FC were strongly modulated by age. Cluster-based permutations tests showed two clusters of PS modulations with age (*p* < 0.001, log-likelihood tests of models with and without age coefficients): with PS decreasing with age for lower frequencies (< 7.7 Hz) and increasing for higher frequencies (> 10.6 Hz). Note that opposite trends in both frequency ranges are expected given the use of relative power. Comparing ASD to NT, we did not find any significant group effects for PS or FC, neither for mean nor for variance (*p* > 0.05, correcting for multiple comparisons with cluster-based permutation tests within PS and FC, respectively; Table 2).

### Multivariate statistics

We next investigated whether a multivariate combination of PS and FC features across space and frequency could separate ASD from NT using three machine learning approaches: linear SVC, elastic net logistic regression and radial basis function SVC with a nested Boruta feature selection (see Methods). Classification performance was overall poor (accuracy 47–57%, sensitivity 50–54%, and specificity 44–61%). For PS and wPLI, the classification performance with the elastic net classifier was greater than expected by chance (*p* = 0.025 and *p* = 0.009, respectively, Table 3).

**Table 3:** ASD vs NT cross-validation classification performance of PS and FC features (training dataset)

			Linear SVC				Elastic net				Boruta + rbf SVC			
Age groups	Diagnosis	Measure	acc	sens	spec	*p*-val	acc	sens	spec	*p*-val	acc	sens	spec	*p*-val
All	ASD	PS	0.52	0.53	0.51	0.24	**0.56**	**0.52**	**0.59**	**0.025**	0.54	0.54	0.54	0.08
All	ASD	OrthPowCorr	0.48	0.50	0.45	0.81	0.50	0.51	0.49	0.45	0.53	0.52	0.53	0.18
All	ASD	wPLI	0.47	0.50	0.44	0.79	**0.57**	**0.53**	**0.61**	**0.009**	0.53	0.50	0.55	0.19
*Build separate models for adults, adolescents and children*														
Adults	ASD	PS	0.50	0.48	0.51	0.50	0.48	0.45	0.52	0.63	0.49	0.50	0.48	0.49
Adolescents	ASD	PS	0.49	0.47	0.51	0.60	0.50	0.50	0.51	0.50	0.48	0.46	0.50	0.56
Children	ASD	PS	0.53	0.51	0.55	0.29	0.47	0.51	0.44	0.68	0.43	0.45	0.42	0.84
Adults	ASD	OrthPowCorr	0.56	0.63	0.49	0.09	0.50	0.48	0.53	0.44	0.47	0.50	0.45	0.76
Adolescents	ASD	OrthPowCorr	0.45	0.51	0.39	0.78	0.50	0.49	0.52	0.40	0.53	0.60	0.46	0.29
Children	ASD	OrthPowCorr	0.60	0.48	0.71	0.07	0.49	0.47	0.51	0.66	0.46	0.44	0.47	0.77
Adults	ASD	wPLI	0.54	0.63	0.45	0.30	0.50	0.45	0.55	0.52	0.52	0.53	0.52	0.31
Adolescents	ASD	wPLI	0.48	0.44	0.53	0.53	0.44	0.43	0.45	0.87	0.43	0.44	0.42	0.91
Children	ASD	wPLI	**0.64**	**0.54**	**0.73**	**0.02**	**0.60**	**0.62**	**0.59**	**0.013**	0.47	0.36	0.56	0.73
*Use a narrower ASD definition*														
All	ASDn	PS	0.53	0.39	0.61	0.45	0.55	0.55	0.56	0.17	0.56	0.48	0.61	0.15
All	ASDn	OrthPowCorr	0.59	0.18	0.82	0.48	0.50	0.52	0.49	0.42	0.53	0.37	0.62	0.79
All	ASDn	wPLI	0.55	0.22	0.73	0.34	0.48	0.53	0.46	0.44	0.55	0.42	0.62	0.26
*Evaluate other variations of EEG power spectral and functional connectivity algorithms*														
All	ASD	abs. PS	0.51	0.51	0.51	0.36	0.53	0.54	0.51	0.14	0.51	0.51	0.52	0.36
All	ASD	PowCorr	0.47	0.48	0.46	0.81	0.46	0.45	0.47	0.99	0.49	0.49	0.48	0.68
All	ASD	PLV	0.47	0.49	0.45	0.82	0.51	0.50	0.53	0.63	0.52	0.52	0.52	0.28
All	ASD	iCOH	0.48	0.53	0.42	0.81	0.46	0.45	0.48	0.87	0.49	0.51	0.47	0.65
All	ASD	COH	0.46	0.47	0.44	0.91	0.47	0.45	0.49	0.88	0.48	0.49	0.47	0.74

Accuracy (acc), sensitivity (sens), specificity (spec) and the *p* value derived from randomization tests are indicated. Classifiers with *p* < 0.05 are highlighted in bold. abs. PS refers to absolute power spectrum and was obtained in sensor space

### Generalizability: age, ASD definition, alternative PS and FC measures

Our results above depend on specific analysis choices including accounting for age as a covariate, criteria for inclusion in the ASD group and choice of specific PS and FC metrics. We next tested whether our results were robust to these specific choices. First, to account for possible nonlinear age dependencies, we built separate ASD vs. NT classification models for children (6–11 years), adolescents (12–17 years) and adults (18–32 years). Such models could uncover potential age-group specific ASD patterns (e.g., alterations only in adults, or only in children and adults but not adolescents) without committing to a pre-specified age-dependency model. Secondly, we used an alternate and “narrower” ASD definition and restricted the ASD sample to individuals who in addition to the ASD clinical diagnosis also met the ASD threshold on the ADOS-2 and on the ADI-R, following [50]. 51.8% of the ASD participants met this additional criterion (*n* = 77 ASD participants in the training dataset, *n* = 110 ASD participants overall, see Additional file 1: Table S5 for a full description of their clinical and demographic characteristics). Thirdly, the effect of EEG processing pipeline choices was assessed by evaluating absolute rather than relative power and alternate FC algorithms with different underlying assumptions on the nature of the coupling between regions’ activities (PowCorr, COH, iCOH and PLV, as described in Materials and Methods). The results are listed in Table 3. Of note, given the amplitude bias in beamforming source reconstructions, absolute power was estimated in sensor space. Classification performance was in general poor and in line with the main analysis described in the previous section, and only significantly better than expected by chance for the linear SVC and the elastic net model with wPLI in children (*p* = 0.02 and *p* = 0.013, respectively).

### Testing the models in the validation dataset

**Fig. 3:**
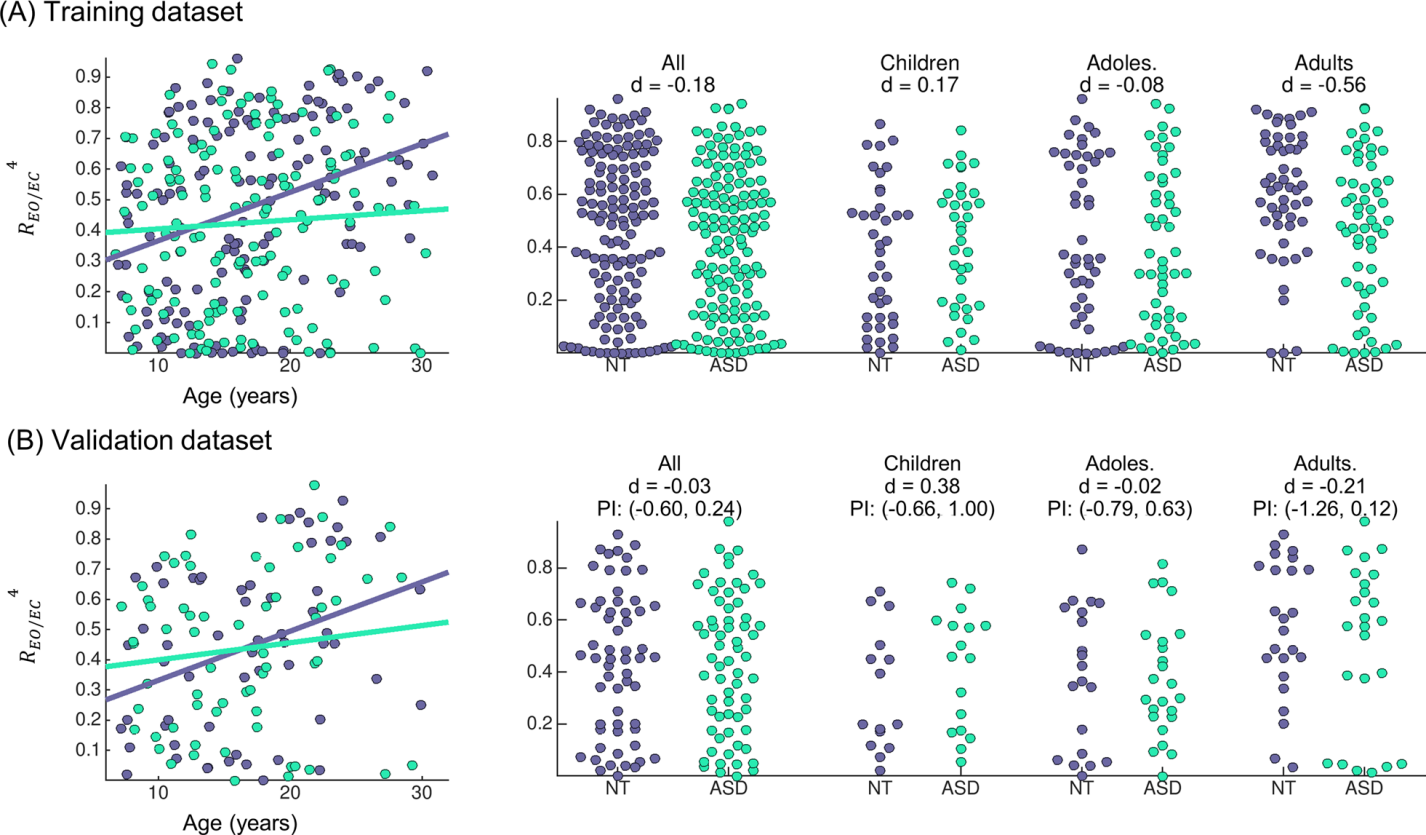
Reactivity to eye opening: distribution and age trends in the training (**A**) and the validation dataset (**B**). The scatter plot of the raw values as a function of age is shown in the left side, along with the regression lines for each group. The distribution of reactivity values for each age group along with the corresponding Cohen’s d effect size is shown on the right side. Typically, *d* ~ 0.20 is considered small and *d* ~ 0.50 a medium effect size. PI indicates the 95% prediction interval from the training dataset to the validation dataset, and it was calculated following [38] based on the training dataset effects and the sample size of both datasets

**Fig. 4:**
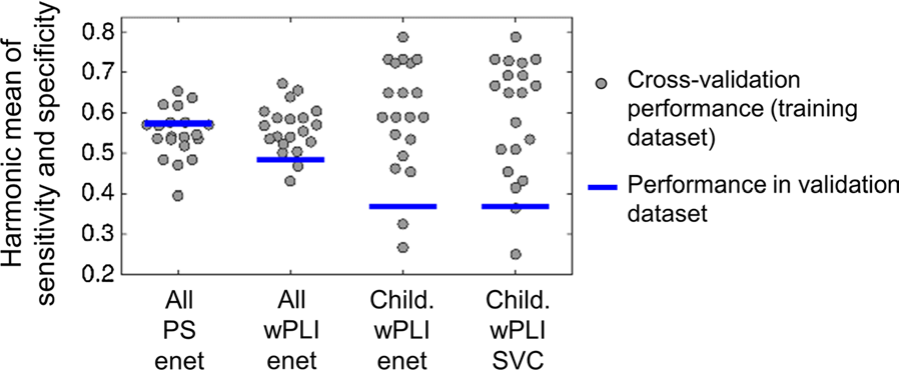
Classification performance of the multivariate models in internal cross-validation and external test in the validation dataset. For each of the four models subjected to testing in the validation dataset, the cross-validation performance of each of the repeated random splits within the training dataset is shown as a gray dot along with the performance in the validation dataset (blue line). All and Child indicate models trained and tested in the whole cohort and children cohort, respectively. enet and SVC represent elastic net and linear support vector classifier models, respectively

Data of 30% of the participants (65 ASD and 59 NT) were hold out from the previous analyses, in order to enable validation of the effects identified in the training dataset (significant group effects for reactivity to eye opening and classification performance for PS and wPLI):

- Reactivity to eye opening was not significantly modulated by group in mean or variance in the validation dataset (*p* > 0.5). Some trends similar to those present in the training dataset can be found in the validation dataset: stronger increase with age in the NT than in the ASD group, and reduced values in adults in ASD compared to NT, see Fig. 3. However, these effects were weak and not significant. Although group effects were not significant, the effect sizes of the case–control alterations across age groups in the validation dataset fell within the 95% prediction intervals of the effects seen in the training dataset, indicating that random variability within the ASD and NT groups alone could account for the deviation of values observed between training and validation dataset. See Fig. 3 for a full overview of effect sizes and prediction intervals across age groups.
- Classification ASD vs NT. The four classification models that produced a significant cross-validation classification performance in the training dataset (*p* < 0.05) were tested in the validation dataset. The results are shown in Table 4. Classification performance was overall poor (accuracy 38–57%) and only above 50% for the elastic net model on PS (*p* = 0.073 compared to randomized versions of the validation dataset). These performance values were in line with those obtained with cross-validation in the training dataset, as shown in Fig. 4.

**Table 4:** Classification performance of the multivariate models tested in the validation dataset

Age groups	Measure	Classifier	acc	sens	spec	S1	*p*-val
All	PS	Elastic net	**0.57**	**0.55**	**0.59**	**0.57**	**0.073**
All	wPLI	Elastic net	0.48	0.46	0.51	0.48	0.68
Children	wPLI	Elastic net	0.38	0.41	0.33	0.37	0.96
Children	wPLI	Linear SVC	0.38	0.41	0.33	0.37	0.96

Accuracy (acc), sensitivity (sens), specificity (spec), S1 and the *p* value comparing the classification performance obtained in the original dataset with that of replicate datasets with randomized group labels is indicated for each model

In summary, all the ASD vs NT alterations found in the training dataset were not significant in the validation dataset (ASD group effects p > 0.05), despite showing similar trends for reactivity to eye opening and PS elastic net model. This could be due to sampling error and to limited statistical power for detecting weak effects in the validation dataset. This is illustrated by the following power considerations. The statistical power to detect an effect size (standardized mean difference) of 0.5 in the validation dataset is 72%, but if this effect is for example restricted to adults only (which could be the case for reactivity to eye opening training dataset), the statistical power is only 18%. This means that there is a high chance of not finding significant effects in the validation dataset even if the true ASD phenotype was close to that found in the training dataset and there was a weak interaction between age and group. More details can be found in Additional file 1.

## Discussion

We investigated differences in resting state EEG parameters between autistic and neurotypical individuals with average to high intellectual abilities from childhood to adulthood in a large and well-controlled cohort (*n* = 212 ASD and *n* = 199 NT). We analyzed various EEG metrics (alpha peak properties, PS, FC) that have been suggested to be altered in ASD. We examined whether the NT and ASD groups differed in in either mean or variance across PS and FC features using univariate testing along with cluster-based permutation tests. We then used machine learning to assess whether combining EEG features could unveil complex patterns separating ASD and NT participants. To test the generalizability of the results, alternative models using a stricter ASD definition and additional PS and FC endpoints were tested, as well as models trained in children, adolescents or adults-only. These analyses were embedded within a train–test approach to enable the testing of various models and underlying hypotheses while minimizing the likelihood of finding false positive results. In the training set, we found significant differences between ASD and NT for three EEG metrics: (1) interaction between age and diagnosis for the reactivity to eye opening with stronger age-related increases in the NT than the ASD cohort (2) significant but rather weak classification performance for PS and wPLI across age groups and for wPLI in children (accuracy of 0.56–0.64, sensitivity 0.52–0.62, specificity 0.59–0.73). The effects in the validation dataset were non-significant, but they were in line with those found in the training dataset (overlapping with the training dataset’s prediction interval or with internal cross-validation performance). This could be explained either by no consistent effects or by consistent albeit weak effects across both datasets.

### Age-dependent maturation of brain rhythms

In line with previous studies of NT developmental trajectories, we observed the expected developmental changes of EEG features, such as age-related increases in alpha peak frequency, relative upper alpha/beta power and average FC [51–54]. This can be seen as a plausibility check for this dataset.

### Our results in the context of the literature

Directly comparing our results with previous publications is complicated by heterogeneity in the methods (e.g., specific EEG parameter extracted, FC metric, regions of interest to estimate pairwise connectivity) and the ASD subpopulation (e.g., age range, IQ, sex). A careful comparison with prior work is nonetheless critical to inform future work with EEG in ASD. Of particular relevance to this effort is O’Reilly et al.’s prior systematic analysis of EEG/MEG FC literature [17]. They evaluated the support for two popular hypotheses in the ASD field—long-range hypoconnectivity and local hyperconnectivity—and concluded that there was relatively strong support for long-range hypoconnectivity in ASD. However, heterogeneity in methods precluded a quantitative meta-analytic confirmation of this phenomenon or of which EEG parameter would best quantify it. Additionally, publications from the largest reviewed dataset [55, 56] enrolling 430 ASD and 554 NT found a mixture of long-range hypo- and hyperconnectivity, suggesting that the long-range hyperconnectivity hypothesis may not fully reflect the complexity of ASD. The authors found an impressive 86% ASD vs. NT classification accuracy, but we cannot test whether their results replicate in the LEAP dataset because their findings are based on a multivariate combination of thousands of features, and the exact combination of features and model weights is not available for download. Although the original study excluded high functioning autism and Asperger [56], the authors found that their original multivariate model also classified NT vs. Asperger with high accuracy [55], so the IQ range of the cohorts do not seem to be causing the discrepancy between their results and ours.

FC in ASD has also been extensively studied in fMRI. Some reviews indicate a consistent pattern of reduced fMRI cortico-cortical connectivity in ASD in adolescents and adults, with an associated and perhaps compensatory increase in short range connectivity [57–59]. In fact, FMRI FC alterations have also been found in the LEAP sample: with both hyperconnectivity in prefrontal and parietal cortices and hypoconnectivity in sensory-motor regions in ASD compared to NT [26]. This pattern of alterations was reproduced in two additional datasets, thereby confirming the robustness of the effects. While both fMRI- and EEG-derived FCs measure aspects of long-distance communication in the brain, the resulting FC values are different, as shown in [60]. Both modalities measure a different type of brain activity (synchronized neuronal activity for EEG, blood oxygenation for fMRI) and have distinct spatial and temporal resolution [61]. Future multimodal analyses comparing directly EEG- and fMRI-derived FC at the single subject level in ASD and NT would be best suited to understand whether the fMRI-derived ASD patterns have an EEG correlate.

Regarding PS, Edgar et al. published the largest study to date [62], with 183 ASD and 121 NT, and provided a testable hypothesis: an interaction between group and age in alpha peak frequency, with strong age-related increases in the NT group and no significant age effects in the ASD group. Within the LEAP dataset, we found age-related increases for both groups. Both datasets have a similar design (cross-sectional, similar age range, no lower IQ participants), so any difference in results is more likely to reflect other factors, such as differences in recruitment, and other more nonspecific effects (e.g., site effects or sampling variability). However, LEAP results for the ASD group are not far from the prediction interval of Edgar et al.: They found *r* = 0.10 in the ASD group, which according to [38] results in a prediction interval for LEAP of [− 0.10, 0.30] and that is close to the value we recovered (*r* = 0.33). For the NT group, our results converge with Edgar and colleagues: They found *r* = 0.57, which leads to a prediction interval [0.41 0.72] which in turn comprises the value obtained in LEAP (*r* = 0.44).

### Lack of a significant effect is not demonstrating no effect

It is important to highlight that a lack of significant effects is different from demonstrating that there are no group differences. Although we had access to a large cohort and would have consequently greater sensitivity to detect core ASD patterns than most previous studies, it is possible and indeed even plausible that there are differences between ASD and NT with smaller effect sizes that we may not have detected. That said, our work suggests that possible differences in resting state EEG features between ASD (IQ > 75) and NT have relatively low effects sizes, which may limit their utility.

We used statistical power simulations to illustrate and quantify the impact of sample size. Accordingly, our statistical power to detect a true alteration of effect size of 0.3 would be 62% in the training dataset, which means that there is a 38% chance of having missed such an effect. In the training dataset, we found a significant interaction between age and group in the reactivity to eye opening, with stronger age-related increases in NT than ASD and strongest different between both groups for the adult cohort (ES =  − 0.57). The ES in the training was − 0.21, which was in the 95% prediction interval from the training dataset, and there is therefore no evidence for discrepancy between both effects. In fact, our statistical power to detect ASD alterations of effect size 0.5 in adults only is 18%, indicating that the validation dataset is not well suited for testing such small age-group specific effects and the need for a bigger cohort to fully assess the reproducibility of these findings.

For multivariate statistics, we found weak classification performances in the training dataset (cross-validation accuracies 0.56–0.64), and in the validation dataset the PS classifier had an accuracy of 0.57 along with a trend for statistical significance (*p* = 0.073). Although this classification pattern may reach significance in a bigger validation dataset, the performance in the training and validation dataset suggests that this is a rather weak classifier which may not have very useful clinical applications. Additionally, machine learning techniques often require a large sample size to achieve good predictive performance [63, 64]. A minimum of 10–20 observations (our in our case, subjects) per variable has been suggested for reliable performance of machine learning methods [65, 66]. Our EEG PS and FC matrices have a high dimensionality, but these variables are correlated and most of their variance is captured in a much smaller number of components (160–389 for PS and FC). Accordingly, future studies with at least 1500 individuals could be therefore better suited to boost the performance of the classifiers. Additionally, although we chose three different and popular state of the art machine learning approaches, it is conceivable that other approaches would be more suited to the problem and to a potential true ASD pattern of alterations. We therefore cannot exclude that with more data and/or other algorithms a better ASD vs. NT separation can be achieved.

### Heterogeneity and possible subgroups in ASD

The heterogeneity of ASD could underlie the lack of significant ASD vs NT differences. ASD has diverse clinical presentations and is in turn thought to be driven by diverse biological substrates [2, 67, 68]. In fact, ASD is common in genetic neurodevelopmental disorders that are characterized by specific and different, sometimes even “opposite” neurobiological and circuitry alterations. It may well be that idiopathic ASD could be split into subgroups of homogeneous neurobiology that substantially differ [3, 68] and possibly even deviate from NT in opposite directions. This could mean for EEG features that the subgroups would change in various directions and the mean might not differ from NT unless appropriate subgrouping is performed. If this were true, it may manifest as increased variance in the ASD group compared to the NT group. We investigated this possibility in the univariate analysis, but could not find any support for the idea that heterogeneous subgroups may drive differential variance in the ASD population as a whole, as compared to NT. Of note, we did not perform clustering in the ASD EEG PS and FC or attempt to find ASD subgroups, but rather focused on the variance of PS and FC measures in ASD compared to NT. Previous studies have used unsupervised statistical learning to identify clusters of ASD subjects based on clinical, behavioral or biological measures [69–71]. Additionally, some studies suggest that differences in brain structure and function in people with ASD are idiosyncratic [72–75]: They are peculiar to an individual person with ASD rather than following a group trend. In this view, each ASD individual could deviate from NT in a different set of features, and no substantial increase in the ASD group variance would be expected for individual PS or FC measures.

### Other measures of brain activity and event-related potentials may provide more information on ASD pathophysiology

Paradigms and brain activity measurements other than resting state EEG may be more sensitive to capture ASD-specific brain circuitry alterations. Indeed, significant case–control effects have been found in previous analyses of resting state fMRI and task EEG in the baseline LEAP recordings. First, as mentioned earlier in the discussion, reproducible fMRI FC alterations have been found in the LEAP cohort [26]. Second, [76] showed an increase in the latency of the N170 component of the event-related potential to faces in ASD compared to NT in the LEAP cohort. The modulation of brain activity during tasks that challenge functions that are often altered in ASD may shed more light onto the ASD-specific circuitry than resting state activity. Interestingly, here we found trends for an effect in the reactivity to eye opening, which is a very basic sensory response measure. Other more elaborated EEG paradigms tapping into elements of sensory or social processing linked to the ASD symptomatology could show stronger effects [77–79].

## Limitations

We cannot draw a firm conclusion on whether the effects on reactivity to eye opening and PS found in the training dataset are reproducible. These effects were weak and the validation dataset is underpowered to confirm them. We have addressed this by estimating prediction intervals and statistical power, as detailed in Results section. Additionally, this is a cross-sectional study and longitudinal data would enable a better estimation of the developmental trajectories of ASD and NT subjects and would lead to more power to detect subtle differences. Finally, here we have exclusively focused on case–control comparisons, but it is possible that other analyses assessing clustering or dimensional correlations with symptom severity could reveal patterns of PS or FC linked to ASD-related symptomatology. Moreover, in this study, we only investigated individuals with an IQ > 75. We opted for this approach because although the LEAP sample includes some individuals with an IQ below 75, they are only included in the adolescent and adult groups in a subset of sites, and we did not want to introduce confounding relations between variables. However, this means that our results may not generalize to people with ASD and intellectual disability or to syndromic forms of ASD. In fact, rare genetic neurodevelopmental disorders characterized by intellectual disability and a symptomatic overlap with ASD such as Fragile X, Angelman and Dup15q syndromes frequently have characteristic resting state EEG features [80–82].

## Conclusions

We found no differences between ASD and NT in resting state EEG that reproduced significantly in the validation dataset, despite trends for reactivity to eye opening and PS. Importantly, alpha peak parameters, PS and FC showed strong and expected age-related maturation from childhood to adulthood in ASD and NT cohorts, demonstrating good data quality and validity of the analytical approaches. In sum, this could indicate, that, across brain rhythms, local and long-range synchronization in ASD (IQ > 75) may overlap largely with the NT distribution. Additionally, no evidence for increased heterogeneity in ASD was found, since the modeled variance in both groups did not differ significantly. Future work could focus on establishing the within-subject developmental trajectory in longitudinal studies, directly targeting the ASD heterogeneity with clustering techniques or evaluating further EEG metrics (e.g., complexity) to further test for specific signatures in the EEG of individuals with ASD.

## Supplementary Information

**Additional file 1.** Supplementary Material. Supplementary tables and figures.

## Data Availability

Data collected in EU-AIMS LEAP are stored and curated at the central EU-AIMS database at the Pasteur Institute in Paris. The database is open to members of the wider scientific community upon request and submission of a paper and data analytic proposal.

## Abbreviations

ADI-R: Autism Diagnostic Interview-Revised
ADOS-2: Autism Diagnostic Observation Schedule, 2nd edition
ASD: Autism spectrum disorder
COH: Coherence
EEG: Electroencephalography
FC: Functional connectivity
iCOH: Imaginary coherence
IQ: Intelligence quotient
LME: Linear mixed effects
MRI: Magnetic resonance imaging
NT: Neurotypical
orthPowCorr: Orthogonalized power correlations
PLV: Phase locking value
PowCorr: Direct power correlations
PS: Power spectrum
ROI: Region of interest wPLI: Weighted phase lag index
