# Supplementary material for: Resting state EEG power spectrum and functional connectivity in autism: a cross-sectional analysis
Source: Mol Autism. 2022 May 18;13:22. doi: 10.1186/s13229-022-00500-x (PMC9118870; doi:10.1186/s13229-022-00500-x)
Supplement: Supplementary file 1 — Additional file 1. Supplementary Material. Supplementary tables and figures. [file 13229_2022_500_MOESM1_ESM.docx]

**Additional File 1**

# Supplementary Tables

Table S1. Overview of clinical and demographic characteristics of the excluded participants. Refer to Table 1 of the main manuscript for the equivalent information on the included participants. The p-values indicate the result of the statistical test comparing the included and excluded participants, for each diagnosis separately. P-values were obtained with t-test for continuous variables and with Fisher’s exact test for categorical variables. Refer to Table 1 for more information on the abbreviations and nomenclature.

|  | discarded ASD | discarded TD | p-value ASD group | p-value TD group |
| --- | --- | --- | --- | --- |
| n | 30 | 12 |  |  |
| Age (years) | 16.0 ± 8.0 | 17.1 ± 7.0 | 0.58 | 0.88 |
| Child / Adol / Adult | 13/5/12 | 3/4/5 |  |  |
| IQ | 100.2 ± 14.7 | 106.4 ± 9.9 | 0.18 | 0.7 |
| Sex (M/F) | 26/4 | 7/5 | 0.12 | 0.55 |
| ADI social | 17.0 ± 7.5 (n=29) |  | 0.22 |  |
| ADI communication | 14.1 ± 5.6 (n=29) |  | 0.15 |  |
| ADI RRB | 4.4 ± 2.3 (n=29) |  | 0.62 |  |
| ADOS Social Affect CSS | 5.5 ± 3.0 (n=30) |  | 0.42 |  |
| ADOS RRB CSS | 5.9 ± 3.2 (n=30) |  | **0.013** |  |
| ADOS Total CSS | 5.4 ± 3.1 (n=30) |  | 0.64 |  |
| VABS | 68.8 ± 16.0 (n=27) | 94.5 ± 43.1 (n=2) | **0.059** | 0.36 |
| SRS2 | 92.3 ± 26.5 (n=27) | 30.8 ± 12.9 (n=11) | 0.22 | 0.25 |
| Medication (%) | 18.5% (n=27) | 0.0% (n=11) | 0.083 | 1.0 |

Table S2. Demographics of the training dataset:

|  |  | n | Age (years) | Child. / Adol. / Adults | IQ | Sex M/F | ADI | | | VABS |
| --- | --- | --- | --- | --- | --- | --- | --- | --- | --- | --- |
|  |  |  |  |  |  |  | social | commun. | RBB |  |
| ASD | all | 147 | 16.8 ± 5.7 | 36/52/59 | 103.6 ± 14.9 | 106/41 | 15.2 ± 6.8 (n=139) | 12.3 ± 5.8 (n=139) | 4.1 ± 2.8 (n=139) | 74.5 ± 14.7 (n=120) |
|  | CIMH | 14 | 15.5 ± 2.3 | 1/11/2 | 103.6 ± 13.8 | 13/1 | 13.9 ± 7.9 (n=13) | 10.2 ± 5.3 (n=13) | 5.2 ± 4.0 (n=13) |  |
|  | KCL | 49 | 16.4 ± 5.1 | 11/18/20 | 101.9 ± 17.1 | 36/13 | 18.0 ± 6.6 (n=47) | 15.5 ± 5.5 (n=47) | 4.9 ± 2.4 (n=47) | 67.6 ± 13.9 (n=45) |
|  | RUNMC | 49 | 15.7 ± 6.4 | 19/15/15 | 103.2 ± 14.3 | 32/17 | 13.3 ± 6.5 (n=48) | 10.7 ± 5.2 (n=48) | 3.0 ± 2.4 (n=48) | 80.5 ± 12.3 (n=46) |
|  | UCBM | 10 | 24.6 ± 2.7 | 0/0/10 | 102.8 ± 15.6 | 8/2 | 12.7 ± 6.9 (n=10) | 10.2 ± 4.1 (n=10) | 5.7 ± 2.7 (n=10) | 73.0 ± 23.3 (n=10) |
|  | UCMU | 25 | 17.3 ± 5.6 | 5/8/12 | 108.0 ± 12.3 | 17/8 | 14.9 ± 5.9 (n=21) | 10.8 ± 6.0 (n=21) | 3.3 ± 2.6 (n=21) | 77.4 ± 9.6 (n=19) |
| NT | all | 140 | 16.8 ± 6.1 | 39/49/52 | 107.4 ± 13.0 | 95/45 |  |  |  | 102.8 ± 10.8 (n=35) |
|  | CIMH | 25 | 14.9 ± 3.8 | 6/15/4 | 109.8 ± 13.7 | 17/8 |  |  |  |  |
|  | KCL | 35 | 18.4 ± 7.3 | 8/9/18 | 110.0 ± 14.9 | 23/12 |  |  |  | 106.4 ± 10.4 (n=8) |
|  | RUNMC | 40 | 14.7 ± 4.3 | 15/17/8 | 102.5 ± 12.7 | 31/9 |  |  |  | 103.7 ± 12.3 (n=17) |
|  | UCBM | 10 | 25.6 ± 2.8 | 0/0/10 | 106.6 ± 11.4 | 6/4 |  |  |  |  |
|  | UCMU | 30 | 16.4 ± 6.1 | 10/8/12 | 109.0 ± 9.1 | 18/12 |  |  |  | 98.5 ± 7.4 (n=10) |

Table S3. Demographics of the validation dataset

|  |  | n | Age (years) | Child. / Adol. / Adults | IQ | Sex M/F | ADI | | | VABS |
| --- | --- | --- | --- | --- | --- | --- | --- | --- | --- | --- |
|  |  |  |  |  |  |  | social | commun. | RBB |  |
| ASD | all | 65 | 16.2 ± 5.7 | 17/24/24 | 105.0 ± 13.6 | 47/18 | 15.6 ± 7.0 (n=63) | 12.9 ± 5.2 (n=63) | 4.4 ± 2.9 (n=63) | 74.2 ± 13.0 (n=54) |
|  | CIMH | 5 | 15.2 ± 3.8 | 1/3/1 | 102.8 ± 11.8 | 2/3 | 13.2 ± 6.8 (n=5) | 13.2 ± 4.8 (n=5) | 6.2 ± 4.4 (n=5) |  |
|  | KCL | 22 | 16.4 ± 5.3 | 4/9/9 | 108.2 ± 15.0 | 15/7 | 18.0 ± 7.8 (n=22) | 15.2 ± 4.5 (n=22) | 5.8 ± 2.9 (n=22) | 69.7 ± 13.6 (n=21) |
|  | RUNMC | 24 | 14.8 ± 5.1 | 8/9/7 | 102.4 ± 13.1 | 21/3 | 15.0 ± 6.0 (n=23) | 12.5 ± 5.1 (n=23) | 2.7 ± 1.8 (n=23) | 75.7 ± 7.6 (n=20) |
|  | UCBM | 3 | 28.4 ± 0.9 | 0/0/3 | 112.9 ± 20.3 | 1/2 | 10.3 ± 4.0 (n=3) | 5.0 ± 3.6 (n=3) | 4.0 ± 1.7 (n=3) | 86.0 ± 30.3 (n=3) |
|  | UCMU | 11 | 16.0 ± 5.9 | 4/3/4 | 103.2 ± 10.6 | 8/3 | 14.7 ± 7.3 (n=10) | 10.8 ± 5.0 (n=10) | 4.2 ± 2.6 (n=10) | 77.2 ± 12.2 (n=10) |
| NT | all | 59 | 16.8 ± 5.9 | 15/20/24 | 109.3 ± 13.4 | 37/22 |  |  |  | 104.4 ± 14.4 (n=13) |
|  | CIMH | 7 | 15.4 ± 3.2 | 1/5/1 | 103.1 ± 12.0 | 5/2 |  |  |  |  |
|  | KCL | 16 | 16.8 ± 6.8 | 4/4/8 | 114.9 ± 11.9 | 8/8 |  |  |  | 113.0 ± 12.1 (n=3) |
|  | RUNMC | 18 | 15.2 ± 4.6 | 6/8/4 | 102.1 ± 15.4 | 11/7 |  |  |  | 101.7 ± 15.8 (n=7) |
|  | UCBM | 5 | 23.6 ± 2.2 | 0/0/5 | 115.8 ± 8.0 | 3/2 |  |  |  |  |
|  | UCMU | 43 | 16.7 ± 6.3 | 14/11/18 | 110.3 ± 9.3 | 28/15 |  |  |  | 99.3 ± 8.7 (n=13) |

Table S4. Overview over the employed classification models. The scikit-learn function (Pedregosa et al., 2011) employed to build the classification model and the associated hyperparameter tuning range are indicated.

| Name | nested feature selection | core classifier | nested hyperparameter tuning |
| --- | --- | --- | --- |
| linSVC | none | linear C-Support Vector Classification (*sklearn.svm.SVC*) | C: [1e-5 to 1e2] |
| elasticnet | none | Elastic net logistic regression (*sklearn.linear_model.SGDClassifier*) | alpha: [1e-3 to 1e2] l1_ratio: [0 to 1] |
| boruta+rbfSVC | Boruta (*boruta_py* , https://github.com/scikit-learn-contrib/boruta_py) | Radial Basis Function (RBF) kernel C-Support Vector Classification (*sklearn.svm.SVC*) | C: [1e-5 to 1e3] gamma:1/Nfeat*[1e-3 to 1e3] |

Table S5. Overview of clinical and demographic characteristics of the ASD participants that meet the ASD threshold on both ADOS and ADI-R (following Risi et al., 2006).

|  |  | n | Age (years) | Child. / Adol. / Adults | IQ | Sex M/F | ADI | | | VABS |
| --- | --- | --- | --- | --- | --- | --- | --- | --- | --- | --- |
|  |  |  |  |  |  |  | social | commun. | RBB |  |
| ASD | all | 110 | 16.4 ± 5.4 | 24/48/38 | 103.5 ± 14.9 | 84/26 | 17.9 ± 5.7 (n=110) | 14.0 ± 5.0 (n=110) | 4.6 ± 2.7 (n=110) | 72.1 ± 14.3 (n=95) |
|  | CIMH | 10 | 15.3 ± 3.1 | 1/7/2 | 104.7 ± 15.0 | 8/2 | 17.1 ± 4.9 (n=10) | 11.7 ± 4.2 (n=10) | 5.0 ± 3.8 (n=10) |  |
|  | KCL | 43 | 16.1 ± 4.8 | 9/19/15 | 103.3 ± 16.4 | 34/9 | 20.5 ± 5.4 (n=43) | 17.0 ± 4.4 (n=43) | 5.7 ± 2.4 (n=43) | 67.2 ± 13.8 (n=42) |
|  | RUNMC | 32 | 14.9 ± 5.5 | 11/14/7 | 101.3 ± 13.5 | 25/7 | 16.1 ± 5.6 (n=32) | 12.9 ± 4.5 (n=32) | 3.3 ± 2.4 (n=32) | 75.3 ± 7.7 (n=30) |
|  | UCBM | 8 | 25.9 ± 2.0 | 0/0/8 | 105.5 ± 20.8 | 5/3 | 14.4 ± 5.2 (n=8) | 10.6 ± 2.9 (n=8) | 4.6 ± 2.3 (n=8) | 78.9 ± 30.5 (n=8) |
|  | UCMU | 17 | 16.4 ± 4.6 | 3/8/6 | 106.4 ± 10.6 | 12/5 | 16.7 ± 5.0 (n=17) | 11.6 ± 5.2 (n=17) | 4.1 ± 2.3 (n=17) | 75.5 ± 9.7 (n=15) |

# Supplementary Methods on FC estimation

Complex time-frequency decompositions of time series were obtained using a Morlet wavelet transform (Tallon-Baudry et al., 1997). This frequency transform accounts for the logarithmic nature of electrophysiological signals (Buzsáki and Draguhn, 2004). Briefly, source time series were convoluted with Morlet wavelets of 0.6 octave frequency resolution (f/σ_f_ = 4.88) and $5\cdot\sigma_{t}$ length, with 90% overlap between windows, for frequencies f = $2^{1:0.15:5}$Hz.

For two given time series $a$ and $b$, we denote their complex wavelet transform at a given frequency $f$ for each epoch e and time window $w$ as: $Z_{a,t,w}=A_{a,e,w}e^{i\varphi_{a,e,w}}$ and$Z_{b,t,w}=A_{b,e,w}e^{i\varphi_{b,e,w}}$. From them, we derive the following FC metrics:

- **Weighted Phase Lag Index (wPLI**) (Vinck et al., 2011)

|  | $\mathrm{wPLI}= \frac{{plsum}^{2}-dbfac}{{wplsum}^{2}-dbfac}$ | (SE2) |
| --- | --- | --- |

Where,

|  | $W_{e,w}=imag\left( Z_{a,e,w}\cdot\mathrm{conj}\left( Z_{b,e,w} \right) \right)$ | (SE3) |
| --- | --- | --- |
|  | $plsum=\sum_{e} \sum_{w} W_{e,w}$ | (SE4) |
|  | $wplsum= \sum_{e} \sum_{w} \left\vert W_{e,w} \right\vert$ | (SE5) |
|  | $dbfac= \sum_{e} \sum_{w} {W_{e,w}}^{2}$ | (SE6) |

The wPLI index estimated here actually corresponds to the debiased estimator of the *squared* WPLI

- **Phase Locking Value (PLV)** (Pereda et al., 2005)

|  | $\mathrm{PLV}= \left\vert\frac{1}{N_{e}}\sum_{e} \frac{1}{N_{w}}\sum_{w} e^{i\left( \varphi_{a,e,w}-\varphi_{b,e,w} \right)} \right\vert$ | (SE1) |
| --- | --- | --- |

- **Coherence and imaginary coherence (Coh and iCoh)** (Nolte et al., 2004)

The complex coherence is defined as:

|  | $\mathrm{CompCoh}= \frac{\sum_{e} X_{ab,e}}{\sqrt{\sum_{e} X_{aa,e}\cdot\sum_{t} X_{bb,e}}}$ | (SE8) |
| --- | --- | --- |

where $X_{ab,e}$ is the cross-spectrum of $a$ and $b$ at epoch $e$ (and frequency $f$)

|  | $X_{ab,e}=\frac{1}{N_{w}}\sum_{w} Z_{a,e,w}\cdot\mathrm{conj}\left( Z_{b,e,w} \right)$ | (SE9) |
| --- | --- | --- |
| and |  |  |
|  | $X_{aa,e}=\frac{1}{N_{w}}\sum_{w} Z_{a,e,w}\cdot\mathrm{conj}\left( Z_{a,e,w} \right)$ | (SE10) |
|  | $X_{bb,e}=\frac{1}{N_{w}}\sum_{w} Z_{b,e,w}\cdot\mathrm{conj}\left( Z_{b,e,w} \right)$ | (SE11) |

Then, the plain coherence is defined as

|  | $\mathrm{Coh}= \left\vert\mathrm{CompCoh} \right\vert$ | (SE12) |
| --- | --- | --- |

And the imaginary coherence as:

|  | $\mathrm{iCoh}=\left\vert\mathrm{imag}\left( \mathrm{CompCoh} \right) \right\vert$ | (SE13) |
| --- | --- | --- |

- **Direct and orthogonalized power correlations (PowCorr and orthPowCorr)** (Hipp et al., 2012)

|  | $\mathrm{PowCorr}= \mathrm{corr}\left( log({A_{a,e,w}}^{2}),log({A_{b,e,w}}^{2}) \right)$ | (SE14) |
| --- | --- | --- |

where corr() denotes the Pearson correlation coefficient

|  | $\mathrm{OrthPowCorr}= \frac{1}{2}\left[ corr\left( log({A_{a,e,w}}^{2}),{{log(A}_{b\perp a,e,w}}^{2}) \right)+corr\left( {{log(A}_{a\perp b,e,w}}^{2}),{{log(A}_{b,e,w}}^{2}) \right) \right]$ | (SE15) |
| --- | --- | --- |

where $A_{b\perp a,e,w}$ is the amplitude complex time series obtained by orthogonalizing $Z_{b,t,w}$ to $Z_{a,t,w}$.

|  | $A_{b\perp a,e,w}= \left\vert\mathrm{imag}\left( Z_{b,t,w}\frac{\mathrm{conj}\left( Z_{a,t,w} \right)}{\left\vert Z_{a,t,w} \right\vert} \right)\frac{i\left( Z_{a,t,w} \right)}{\left\vert Z_{a,t,w} \right\vert} \right\vert$ | (SE14) |
| --- | --- | --- |

And similarly $A_{a\perp b,e,w}$ is the amplitude complex time series obtained by orthogonalizing $Z_{a,t,w}$ to $Z_{b,t,w}$.

Estimates of phase consistency (wPLI, PLV, Coh and iCoh) are biased measures. I.e. even in the absence of any phase consistency, the measures provide positive values that decay with the number of analysis windows used. Given varying amount of usable data for different subjects, using all data available would lead to unwanted bias-related variance that in the worst case could differ between groups to compare. To avoid this problem but at the same time to use as much data as possible for deriving accurate phase-relation measures we took the following approach:

In the previous equations (SE1-11), summation over epochs are computed over $N_{e}$= 15 2.5-second epochs. These 15 epochs are selected randomly from all the clean epochs from a given subject and condition, and this process is repeated 100 times. The final wPLI, PLV, Coh and iCoh estimates correspond to the average value over the 100 randomizations. 2.5 seconds were selected to fit one Molet Wavelet of the lowest frequency analyzed. The threshold of $N_{e}$= 15 epochs was selected as the lower bound of data available for most subjects (i.e. 440 of 453 subjects had at least 15 epochs of 2.5 second long data sections). The random sampling allowed to take all data into account for subjects that had more than the minimum data.

Since correlation coefficients are not biased by sample size, correlation coefficients in (SE14) and (SE15) are computed using all available clean epochs and wavelet windows for a given subject and condition.

# Supplementary section: Site effects

To investigate the significance of site effects on EEG parameters, we used log-likelihood tests between LME models with and without site effects. For the alpha peak parameters, site effects were significant (p<0.05) for reactivity to eye opening and eyes open alpha power, and non-significant for eyes closed alpha power and alpha peak frequency. The significance of site effects for the continuous PS and FC parameters is summarized in Table S6. Site effects impacted significantly a higher fraction of PS than FC features, particularly during the eyes open condition. These site effects can be caused by different factors, including recording setup and conditions or differences between participants (NT or ASD) across sites. We note that, even though the protocol for LEAP acquisition was standardized (common electrode layout across sites, same design of alternating 30s resting state blocks), there may be systematic differences across sites such as light levels or recording environment. We can also wonder if group effects differed across sites. We assessed this for alpha reactivity (which is the only EEG parameter that showed significant group effects in the univariate analysis) and we found no significant interaction between site and group (log-likelihood test p>0.9).

Table S6. Fraction of EEG parameters with significant site effects (p<0.05).

| EEG parameter type | Eyes open | Eyes closed |
| --- | --- | --- |
| PS | 5110 / 9855 – 52% | 1385 / 9855 = 14% |
| FC – wPLI | 1237 / 33075 – 4% | 820 / 33075 – 2% |
| FC – orthPowCorr | 3904 / 33075 – 12% | 2580 / 3305 - 8% |

# Supplementary section: Medication effects

We investigated whether the medication usage affected EEG parameters with significant group effects reported in the main manuscript.

- Reactivity to eye opening did not differ significantly between ASD subjects receiving a psychoactive medication and those who did not (p = 0.67, log-likelihood ratio comparing a model with age, sex and IQ as fixed effects with another model with an additional medication factor).
- For the four multivariate classification models that produced a significant cross-validation classification performance in the training dataset, we evaluated their classification performance for the ASD medication subgroups. The results are displayed in Figure S1. There was no systematic effect of medication, but cross-validation performance differed between medication subgroups for the elastic net models trained on the power spectrum and wPLI features: higher classification performance for medicated than non-medicated participants for the PS model, and opposite effects for the wPLI model (p < 0.01).

It is important to note that different psychoactive medications may have different effects on brain function, so this is only a preliminary response to the question.


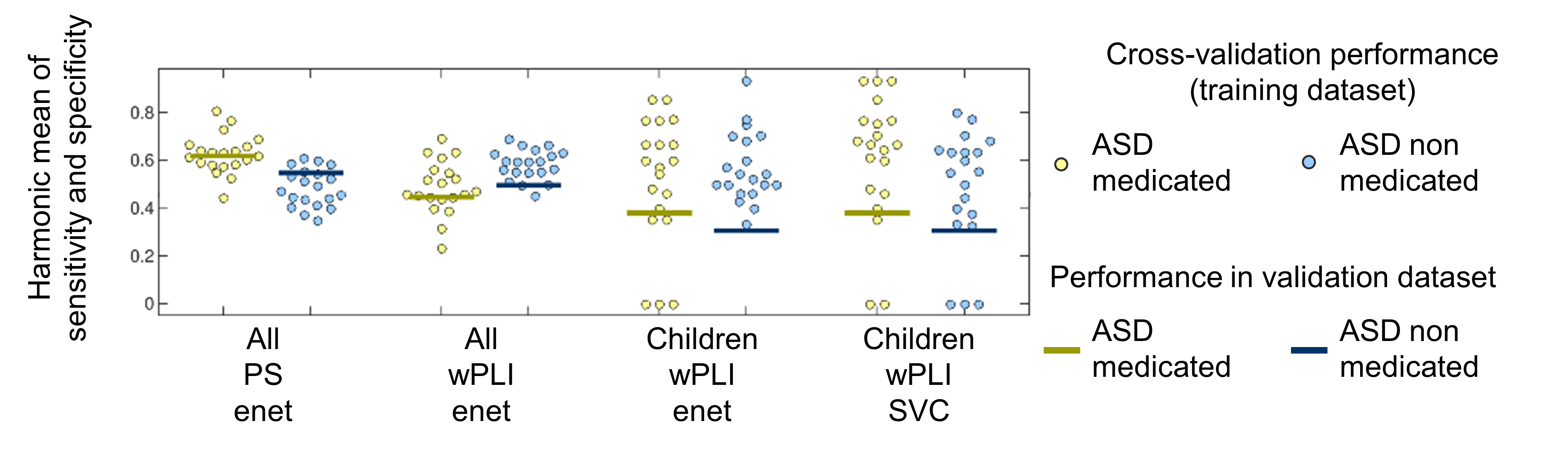


Figure S1. Classification performance on ASD medicated and non-medicated participants of the four multivariate models reported in the main manuscript. This figure is equivalent to Figure 4 of the main manuscript, but assessing the classification performance separately for the medicated and the non-medicated ASD subgroups.

# Supplementary section: Multivariate analysis without prior regression

We performed the classification analysis (cross-validation within training dataset), without the prior regression of y ~ 1 + age + sex + IQ + (1|site). We instead used the raw PS and FC features as input to the classification models. The results are shown in Table S7. They are in line with the results obtained in the main analysis: strongest performance for wPLI and elastic net model. However, the classification performance (as measured with S1, sensitivity or specificity) is slightly worse than for the main analysis. Consequently, for this dataset, the regression step has not been detrimental to the classification performance.

Table S7. ASD vs NT cross-validation classification performance of PS and FC features (training dataset), using the raw PS and FC features (without prior regression). Accuracy (acc), sensitivity (sens), specificity (spec) and the p-value derived from randomization tests are indicated.

|  |  |  | linear SVC | | | | elastic net | | | | boruta + rbf SVC | | | |
| --- | --- | --- | --- | --- | --- | --- | --- | --- | --- | --- | --- | --- | --- | --- |
| age groups | diagnosis | measure | acc | sens | spec | p-val | acc | sens | spec | p-val | acc | sens | spec | p-val |
| all | ASD | PS | 0.51 | 0.5 | 0.53 | 0.36 | 0.52 | 0.52 | 0.52 | 0.2 | 0.5 | 0.51 | 0.5 | 0.48 |
| all | ASD | OrthPowCorr | 0.52 | 0.57 | 0.47 | 0.35 | 0.49 | 0.47 | 0.52 | 0.98 | 0.5 | 0.49 | 0.5 | 0.5 |
| all | ASD | wPLI | 0.49 | 0.55 | 0.43 | 0.66 | **0.56** | **0.55** | **0.56** | **0.022** | 0.55 | 0.52 | 0.57 | 0.11 |

# Supplementary section: statistical power

Although we analyzed a large sample, our analyses may have missed true ASD vs. NT differences. This would be in particular the case if these effects were inconsistent across the ASD population and only present in smaller ASD subgroups. To explore this this more quantitatively we evaluated the statistical power of detecting group effects in our sample. For this purpose, we focused on the reactivity to eye opening and four scenarios: (1) homogeneous effects across all ASD participants (2) effects only in adults (3) effects only in males (4) effects only in adult males.

We first assessed the statistical power of detecting a significant ASD vs. NT group effect in mean, when simulating a range of true group effect sizes between 0 and 1. In each case, 5,000 randomizations of the original dataset were performed. Values for each randomization were defined as yrand = yfit + simulated_group_effects + randomerror, where yfit is the prediction of the model y ~ age + sex + iq + (1|site), simulated_group_effects contains the simulated fixed effect for group, and randomerror is pooled from a normal distribution of mean zero and standard deviation equal to that of the original model’s residual error ($SD_{residuals})$. The effect size is determined as the standardized mean difference, using the overall standard deviation of the population $SD_{tot}$ (without removing the variance generated by age, sex, iq and site).

For group effects in variance, we followed a similar approach using yrand = yfit + randomerror, where the standard deviation of the random error is $SD_{residuals}$ for NT subjects and $\sqrt{z^{2}{SD_{tot}}^{2}-({SD_{tot}}^{2}- {SD_{residuals}}^{2})}$ for ASD subjects (z is the ratio between the simulated standard deviation of the ASD and NT groups)

An overview of the results is shown in Figure S1. Although there is a 98% power of detecting a homogeneous effect size of 0.5 in the training dataset, the statistical power is reduced to 50% if this effect is only present in adults, and to 32% if it is only present in adult males. When using the validation dataset, these values are reduced to 72%, 30% and 18% respectively. Regarding differences between the variance of the ASD and NT groups, the statistical power of detecting a true change in the SD of the ASD of +15%, +20% and +30% is 52%, 72% and 95%, respectively (see Figure S2)


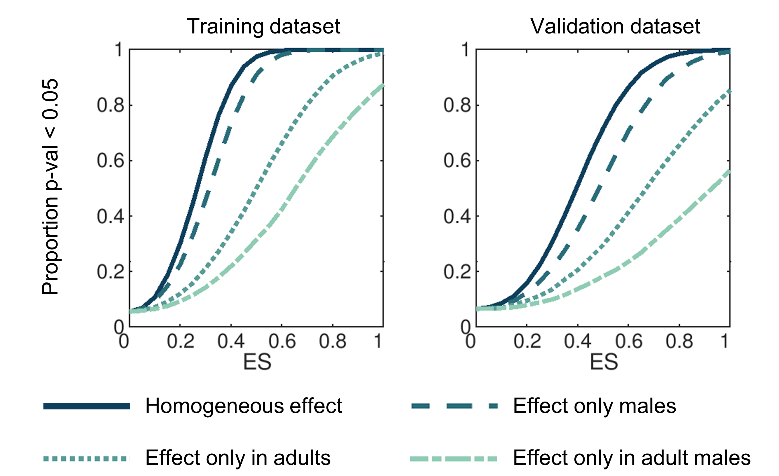


Figure S2. Statistical power to detect significant group effects in the reactivity to eye opening in the training and validation datasets. Four different scenarios are considered: (1) homogeneous effects across all ASD participants (2) effects only in adults (3) effects only in males (4) effects only in adult males. The y-axis shows the fraction of randomizations leading to a group effect of p-value < 0.05. ES = effect size.


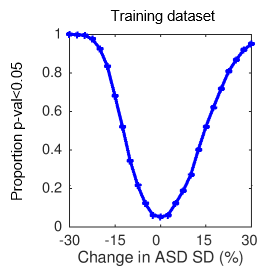


Figure S3. Statistical power to detect significant group effects in variance in the reactivity to eye opening in the training dataset. The y-axis shows the fraction of randomizations leading to a group effect of p-value < 0.05.

REFERENCES

Buzsáki, G., Draguhn, A., 2004. Neuronal oscillations in cortical networks. Science 304, 1926–1929.

Hipp, J.F., Hawellek, D.J., Corbetta, M., Siegel, M., Engel, A.K., 2012. Large-scale cortical correlation structure of spontaneous oscillatory activity. Nat. Neurosci. 15, 884–890. https://doi.org/10.1038/nn.3101

Nolte, G., Bai, O., Wheaton, L., Mari, Z., Vorbach, S., Hallett, M., 2004. Identifying true brain interaction from EEG data using the imaginary part of coherency. Clin. Neurophysiol. 115, 2292–2307. https://doi.org/10.1016/j.clinph.2004.04.029

Pereda, E., Quiroga, R.Q., Bhattacharya, J., 2005. Nonlinear multivariate analysis of neurophysiological signals. Prog. Neurobiol. 77, 1–37. https://doi.org/10.1016/j.pneurobio.2005.10.003

Tallon-Baudry, C., Bertrand, O., Delpuech, C., Pernier, J., 1997. Oscillatory γ-band (30–70 Hz) activity induced by a visual search task in humans. J. Neurosci. 17, 722–734.

Vinck, M., Oostenveld, R., van Wingerden, M., Battaglia, F., Pennartz, C.M.A., 2011. An improved index of phase-synchronization for electrophysiological data in the presence of volume-conduction, noise and sample-size bias. NeuroImage 55, 1548–1565. https://doi.org/10.1016/j.neuroimage.2011.01.055
